# Novel e-Health Applications for the Management of Cardiometabolic Risk Factors in Children and Adolescents in Greece

**DOI:** 10.3390/nu12051380

**Published:** 2020-05-12

**Authors:** Athanasia Tragomalou, George Moschonis, Yannis Manios, Penio Kassari, Ioannis Ioakimidis, Christos Diou, Leandros Stefanopoulos, Eirini Lekka, Nicos Maglaveras, Anastasios Delopoulos, Evangelia Charmandari

**Affiliations:** 1 Division of Endocrinology, Metabolism and Diabetes, First Department of Pediatrics, National and Kapodistrian University of Athens Medical School, “Aghia Sophia” Children’s Hospital, 11527 Athens, Greece; peniokassari@gmail.com (P.K.); evangelia.charmandari@googlemail.com (E.C.); 2 Division of Endocrinology and Metabolism, Center of Clinical, Experimental Surgery and Translational Research, Biomedical Research Foundation of the Academy of Athens, 11527 Athens, Greece; 3 Department of Dietetics, Nutrition and Sport, School of Allied Health, Human Services and Sport, La Trobe University, Melbourne VIC 3086, Australia; G.Moschonis@latrobe.edu.au; 4 Department of Nutrition and Dietetics, Harokopio University of Athens, 70 El Venizelou Avenue, Kallithea, 17671 Athens, Greece; manios@hua.gr; 5 Department of Biosciences and Nutrition, Karolinska Institutet, 17177 Stockholm, Sweden; Ioannis.Ioakimidis@ki.se; 6 Department of Electrical and Computer Engineering, Aristotle University of Thessaloniki, 54124 Thessaloniki, Greece; diou@mug.ee.auth.gr (C.D.); adelo@eng.auth.gr (A.D.); 7 Department of Medicine, Lab of Computing Medical Informatics and Biomedical Imaging Technologies, Aristotle University of Thessaloniki Medical School, 54124 Thessaloniki, Greece; 6.leandros@gmail.com (L.S.); lekka@auth.gr (E.L.); nicmag@med.auth.gr (N.M.)

**Keywords:** childhood obesity, e-Health, registries, big data, algorithms, Pedobesity, cardiometabolic risk factors

## Abstract

Obesity in childhood and adolescence represents a major health problem. Novel e-Health technologies have been developed in order to provide a comprehensive and personalized plan of action for the prevention and management of overweight and obesity in childhood and adolescence. We used information and communication technologies to develop a “National Registry for the Prevention and Management of Overweight and Obesity” in order to register online children and adolescents nationwide, and to guide pediatricians and general practitioners regarding the management of overweight or obese subjects. Furthermore, intelligent multi-level information systems and specialized artificial intelligence algorithms are being developed with a view to offering precision and personalized medical management to obese or overweight subjects. Moreover, the Big Data against Childhood Obesity platform records behavioral data objectively by using inertial sensors and Global Positioning System (GPS) and combines them with data of the environment, in order to assess the full contextual framework that is associated with increased body mass index (BMI). Finally, a computerized decision-support tool was developed to assist pediatric health care professionals in delivering personalized nutrition and lifestyle optimization advice to overweight or obese children and their families. These e-Health applications are expected to play an important role in the management of overweight and obesity in childhood and adolescence.

## 1. Introduction

Obesity in childhood and adolescence represents one of the most challenging public health problems of the 21st century. It is often associated with insulin resistance, dyslipidemia, hypertension, inflammation and endothelial dysfunction, leading to the development of diabetes mellitus type 2 and cardiovascular disease [1,2]. The incidence of obesity has tripled since 1975. According to the World Health Organization (WHO), overweight and obesity are estimated to affect 41 million children younger than 5 years, and more than 340 million children and adolescents aged 5–19 years [3]. In Greece, the prevalence of overweight and obesity is at least 30–35% [4].

Overweight and obesity in childhood and adolescence are associated with significant morbidity and mortality in adulthood [5,6,7,8,9,10]. Obesity accounts for approximately 5% of all deaths worldwide [11], and it may reverse the current declining rate of mortality owing to cardiovascular causes. In addition to the increased morbidity and mortality, overweight and obesity account for a significant increase in public health costs. The global economic impact from obesity is approximately $2.0 trillion USD or 2.8% of the global gross domestic product (GDP), which is almost equivalent to the global impact from smoking or armed violence, war and terrorism [11].

The increasing prevalence of overweight and obesity indicates that the existing health policies are not effective and calls for further advancements in our strategy for the prevention and management of overweight and obesity in childhood and adolescence [11,12]. The need for community-targeted actions has long been recognized, however, it has been hindered by the lack of monitoring, evaluation framework, and methodological ability to objectively quantify the local community characteristics in a reasonable timeframe. Fortunately, technological advancements in e-Health applications, and more specifically, big data analytics, have improved healthcare significantly by facilitating precision and personalized medicine. Furthermore, they allow direct engagement of European citizens in the data collection process, helping to reshape policies at a regional, national and European level.

The aim of this review is to present a range of novel e-Health applications developed and implemented in Greece, in order to provide a comprehensive and personalized plan of action for the prevention and management of overweight and obesity in childhood and adolescence.

## 2. A/The Hellenic National Registry for the Prevention and Management of Overweight and Obesity in Childhood and Adolescence

We used information and communication technologies to develop a web application, the “National Registry for the Prevention and Management of Overweight and Obesity in Childhood and Adolescence”, in order to register online all children and adolescents nationwide, as well as to guide and train Pediatricians and General Practitioners regarding the management of an overweight or obese child and adolescent [13]. Children and adolescents are classified as obese, overweight or of normal body mass index (BMI) according to the International Obesity Task Force (IOTF) cut-off points [14]. This application was developed as part of the Program “Development of a National System for the Prevention and Management of Overweight and Obesity in Childhood and Adolescence” in Greece, it represents part of the Hellenic and European policy to develop and use reliable Patient Registries, and supports interoperability with other infrastructures (i.e., e-Prescription). It was sponsored by the National Strategic Reference Framework (NSRF) 2007–2013 under the Operational Program “Human Resources Development” (EP.AN.A.D) 2007–2013 and was co-funded by the European Social Fund (ESF) and National Funding [13].

Specifically, we developed an electronic medical records file (EMRF) for the electronic documentation of the present, past and family medical history, as well as the clinical examination findings of all children and adolescents. In addition, we developed the therapeutic algorithm files (TAF), which provide detailed information and guidance to pediatricians and general practitioners to allow for the personalized management of each subject. Upon entering the EMRF, each pediatrician or general practitioner has the opportunity to create a new EMRF in order to record a new patient, while he/she is able to view only the EMRFs of the patients under his/her care. In the EMRF, the physician records information on personal and demographic data, such as the social security number, name, surname, birth date and demographic data, the child’s medical history (perinatal history, present and past medical history, nutrition, level of physical activity and exercise, allergies, immunizations, hospitalizations, surgeries, gynaecological history and medical treatments), family history, clinical examination findings (including the anthropometric parameters and arterial blood pressure) and patient consent [15].

The electronic database system (EDS) then automatically calculates the BMI from the data on height and weight, and informs the physician whether the patient has normal or increased BMI. In addition, the corresponding growth chart for BMI appears on the computer screen. Subsequently, the EDS selects the most appropriate therapeutic algorithm file (TAF), which provides a comprehensive and personalized multidisciplinary management plan for the prevention and/or management of overweight and obesity for the patient. The TAF indicates what the initial advice to the patient and his/her family should be; when the physician should reassess the patient; how he/she will manage the patient if there is adequate response to the therapeutic interventions and how if there is no response to the therapeutic interventions despite compliance with those; when he/she will perform laboratory investigations and which ones; when he/she will refer the patient to a pediatric dietician or psychologist; and when he/she will refer the patient to a pediatric endocrinologist or a specialist center with expertise in the management of overweight and obesity [15]. The installation and hosting of the central database have been offered by the Athens Medical Society, and the development was implemented by Datamed SA—a systems integration and consulting services company [15].

To the best of our knowledge, the Hellenic “National Registry for the Prevention and Management of Overweight and Obesity in Childhood and Adolescence” is the first web-based e-Health application in Greece developed to be used by health professionals. It was launched in September 2015 and is accessible by the following URL: http://app.childhood-obesity.gr/. An important distinction of the project was the BRONZE Award in the category “e-Health/H1.1 Digital Applications for Integrated Patient care’’, which was awarded by the Healthcare Business Awards in 2016.

The effectiveness of the interventions suggested by this electronic application was evaluated in our out-patient clinic for the Prevention and Management of Overweight and Obesity, Division of Endocrinology, Metabolism and Diabetes, First Department of Pediatrics, National and Kapodistrian University of Athens Medical School, “Aghia Sophia” Children’s Hospital, Athens, Greece. At present, more than 3,000 children and adolescents are followed up on a regular basis. We demonstrated that this personalized multi-disciplinary management plan is effective in reducing the prevalence of obesity in childhood and adolescence after one year of intervention [15,16].

## 3. B/Development of Intelligent Multi-Level Information Systems and Specialized Artificial Intelligence Algorithms for Personalized Management of Obesity in Childhood and Adolescence

Another innovative e-Health application is part of the Operational Program “Competitiveness, Entrepreneurship & Innovation, EPAnEK 2014–2020” (project code: T1EDK-01386, MIS: 5030543, Acronym: PEDOBESITY) and refers to the development of intelligent multi-level information systems and specialized artificial intelligence algorithms for personalized management of obesity in childhood and adolescence [17]. The program is co-funded by Greece and the European Union, and represents a continuation of the Program “Development of a National System for the Prevention and Management of Overweight and Obesity in Childhood and Adolescence” in Greece. The aim of this project is the continuation of the electronic registration, as well as the prevention, personalized management and monitoring of all children and adolescents with overweight and/or obesity from birth to adulthood in Greece using intelligent information and support systems. To this end, innovative technologies are being used in conjunction with the existing infrastructure and human resources of the primary health care.

**Figure 1:**
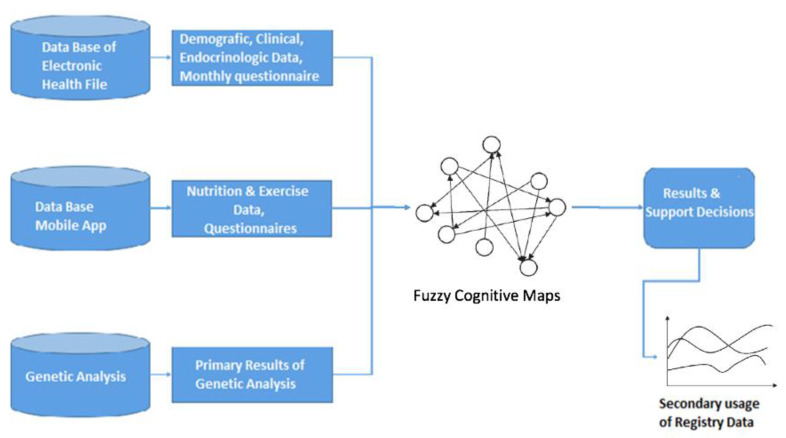
Pedobesity—Development of Intelligent Multi-level Information Systems and Specialized Artificial Intelligence Algorithms for Personalized Management of Obesity in Childhood and Adolescence: The Main Innovative Actions.

The main innovative actions of this application include: (a) collection and analysis of clinical, hematological, biochemical, endocrinologic and genetic data of overweight and obese children and adolescents followed up at our Out-patient Clinic for the Prevention and Management of Overweight and Obesity in Childhood and Adolescence; (b) detection of polymorphisms associated with obesity, diabetes mellitus type 2 and antioxidant capacity; (c) development of a specific obesity-risk algorithm by linking each genotype to the patient’s data, as well as published information on how it affects body weight at clinical or genetic level; and (d) development of interconnected online and mobile applications to integrate the ecosystem of applications for childhood obesity. Applications include the development and expansion of the EMRF of the “National Registry for the Prevention and Management of Overweight and Obesity in Childhood and Adolescence”, patient access subsystem and online support, as well as mobile application for children, adolescents and their parents; and finally (e) the development of an intelligent data management platform (in full interoperability with the “National Registry for the Prevention and Management of Overweight and Obesity in Childhood and Adolescence”) using the innovative methodology of fuzzy cognitive maps and modeling techniques from medical data analysis in order to provide personalized management guidelines (Figure 1).

The Development of Intelligent Multi-Level Information Systems and Specialized Artificial Intelligence Algorithms for Personalized Management of Obesity in Childhood and Adolescence is currently in progress. The goal of this project is to reduce overweight and obesity rates in Greece by at least 20% within 5 years following implementation of all the above applications, thereby reducing the cardiometabolic risk associated with obesity.

## 4. C/Big Data against Childhood Obesity

Over the last decade, the widespread use of personal digital technologies, such as smart mobiles and accelerometers, create new possibilities for the collection of behavioral data objectively. At the same time, the value of using large-scale data (big data) in modern healthcare settings has been widely accepted. Big data methodologies have evolved to the level of processing, analyzing and evaluating a large volume of data, and are expected to restructure public health by shifting the focus of health care to prevention rather than treatment. The development of such methodologies will enable scientists and public health authorities to collect and analyze objective data on the daily behavior of children at high risk of developing overweight and/or obesity, and to modify public health policy strategies at local level accordingly.

A scientific project that is based on these principles is the project entitled “BigO: Big Data against Childhood Obesity” [18], which is a European Union-funded project (http://bigoprogram.eu, Horizon2020, No. 727688). The project is coordinated by the Department of Electrical and Computer Engineering, Aristotle University of Thessaloniki, Greece, it is of four years’ duration, and brings together 13 European partners from Greece, Sweden, Ireland, Spain and the Netherlands. The aim of BigO is to create technological/scientific tools for clarifying the associative dependencies among local living-environmental conditions and individually recorded behavioral patterns that result in increased BMI, focusing on students and age-matched children and adolescents with obesity across different European countries [19,20]. More specifically, BigO aims to evaluate a range of novel technologies for collecting objective measurements of obesogenic behaviors of children and adolescents during their day-to-day life, and analyze them in relation to their local environment.

The planned data collections in BigO are conceptually and practically divided into: (a) the general student population data collection, and (b) the aged-matched clinical population data collection (corresponding to the clinical use of the system). In the BigO project, children become citizen scientists and also help their clinical trajectory by collecting information on their own living environment and personal behavior. This information consists of both objective, passively recorded measurements [such as physical activity levels, sleep recordings and Global Positioning System (GPS)-based analysis of visitation to relevant points-of-interest—i.e., food retailers] and measurements actively uploaded by the users (such as meal pictures and daily mood self-evaluations). The data collection system includes the BigO technology platform, which interfaces with a smartphone and optionally a smartwatch, and records data objectively (using inertial sensors and GPS) for each patient. Data are then transmitted to BigO servers to extract behavioral indicators [21], including: (a) physical activity/exercise, (b) dietary habits, and (c) environmental conditions (urban, socio-economic, and nutritional). Collected data are anonymized and encrypted [22]. Data are stored exclusively in European data centers, which comply with the EU Directive 95/46/EC and the newer EU General Data Protection Regulation (GDPR).

The project intends to: (i) collect individual-level behavioral big data (extracted based on accelerometry and location data) and accompanying qualitative measures (e.g., mood questionnaire); (ii) analyze the collected individual data in conjunction with relevant publicly available community data (data from geographical information systems, public epidemiological databases, official statistics etc.); (iii) create comprehensive population models about the community obesity prevalence matrix, through the association of the quantified local community conditions and the recorded community behavioral patterns; (iv) develop and deploy a technological platform to be used by public authorities to plan health policies against childhood and adolescent obesity; (v) to predict their efficiency in specific communities and to monitor their implementation in near real-time; and (vi) create side-tools for the meaningful use of dataset segments by clinics, schools and school authorities and scientists. The overall aim is to collect and analyze big data on the behavior and living environments of overweight and obese children and adolescents in order for public health authorities to plan and execute effective programs to reduce the prevalence of childhood obesity.

**Figure 2:**
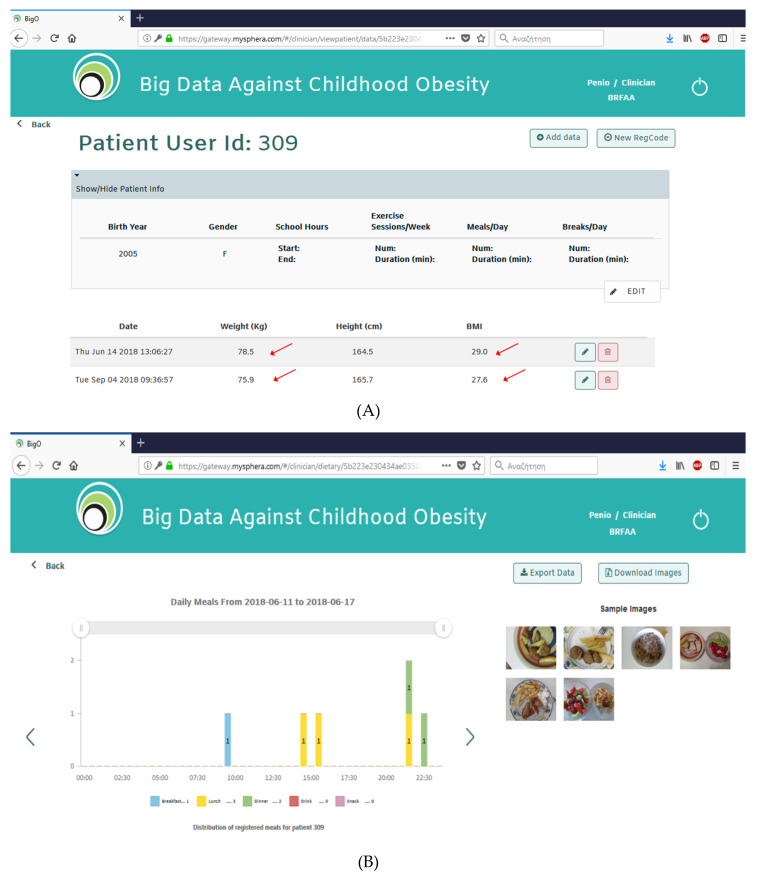

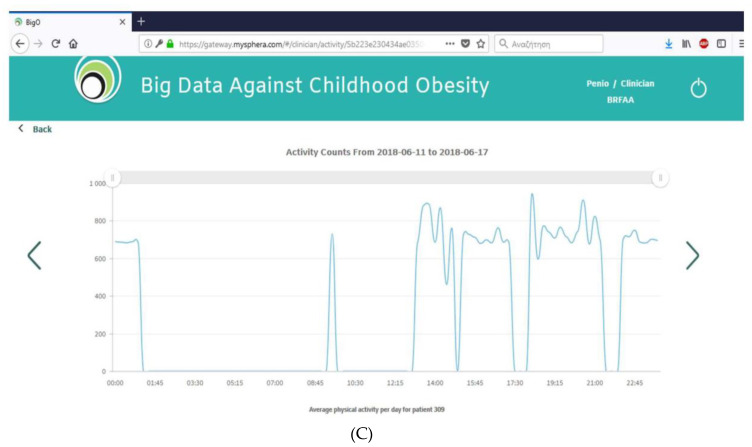
The Big Data against Childhood Obesity Platform: (**A**) decrease in weight and body mass index (BMI); (**B**) photographs of foods consumed; (**C**) the average physical activity per day per week.

The clinical study of this project involves the collection of data provided by children and adolescents followed up at our Out-patient Clinic for the Prevention and Management of Overweight and Obesity. Written informed consent is obtained by parents/guardians in all cases. The study includes overweight and obese children and adolescents, aged 9–18 years, and is divided into three phases. During the first pilot phase (phase 0), which has been completed, 40 children and adolescents (11 males, 29 females) aged 9–18 years used the BigO system for 2 weeks. After 3 months of diet and physical exercise interventions, they were asked to use the system for another 2 weeks. They took photographs of the food they consumed and wore the watch for specific periods during the week (at least 2 weekdays, 1 weekend and 3 nights). Participants were asked to return the watch and complete a system and process evaluation questionnaire. The subjects uploaded a total of 571 meal photographs from their mobile phone camera and recorded 177 days of accelerometry data from the smartphone or smartwatch (Figure 2). Seventy-seven percent (77%) of the participants expressed a positive or neutral opinion when assessing the system.

Phase 1, which has also been completed, included 750 participants (372 males, 378 females) aged 9–18 years, who were instructed to use the system for four consecutive weeks [23]. All subjects uploaded a total of 17,987 meal photographs from their mobile camera and recorded 19,643 days of inertial sensor data from the smartphone or smartwatch (12,354 days of accelerometer data and 7289 days of GPS data). Eighty-eight percent (88%) of the participants expressed a positive or neutral opinion when assessing the system.

Data collection is ongoing (Phase 2), with another 800–1000 patients being scheduled to contribute data till the end of 2020. Similarly, data analysis is underway and is expected to be completed by mid-2021.

## 5. D/Decision Support Tool (DST) for Pediatric Health Care Professionals

In order to assist pediatricians and pediatric endocrinologists to deliver personalized advice on nutrition and lifestyle to children and their families, a computerized decision-support tool (DST) was developed by the Department of Nutrition and Dietetics at Harokopio University, Athens, Greece [24]. The development of the computerized DST is based on decision-tree algorithms, which include five different levels. The first level (level 1) relates to the “assessment of children’s current weight status”, which is based on the documentation of body weight in all subjects from infancy to adolescence, and of the recumbent length in infants and children until the age of 2 years or standing height in all children and adolescents beyond the age of 2 years. The second level (level 2) relates to the “assessment of the likelihood for the future manifestation of obesity in normal-BMI children”, which combines the effect of individual obesity risk factors, including socio-demographic and perinatal factors in order to estimate the likelihood of the development of obesity in the future. For this purpose, the core (Childhood Obesity Risk Evaluation Index) [25] was included as another component of the DST, which represents a simple, easy-to-use, valid score, [26] and provides an estimation of the future likelihood of development of obesity as early as at the age of 6 months. The third level (level 3) refers to the “evaluation of the most appropriate body weight management goal” by using the recommendations of the American Pediatric Association for the prevention and treatment of childhood obesity, which include two weight management pathways: (i) maintenance of body weight, which aims to reduce BMI gradually as a result of the increase in height in growing children; or (ii) reduction of body weight in case that comorbidities (i.e., hyperglycemia, insulin resistance, dyslipidemia, hypertension) co-exist in association with obesity. The fourth level (level 4) relates to the “estimation of children’s dietary energy and macronutrients intake needs”. More specifically, the DST calculates the estimated energy requirements (EER) (in kcals per day) of children and the percent of energy derived from macronutrients, according to the acceptable macronutrient distribution ranges (AMDRs) proposed by the IOM (mathematical formulas provided by the Institute of Medicine) [27] for carbohydrates, fat and protein. Based on all aforementioned information, the DST proposes a “personalized diet and lifestyle optimization advice” (level 5) to children and their families.

The effectiveness of the DST was evaluated through a pilot randomized controlled intervention trial, which included 80 overweight or obese children aged 6–12 years with BMI-for-age ≥ 85th percentile. Subjects were randomized into the control group (CG) and the intervention group (IG). The IG received personalized meal plans and recommendations on lifestyle modification according to the DST, while the CG received general recommendations. Data were collected at baseline and three months later [24]. After three months of intervention, the IG demonstrated a statistically significant change in dietary fiber and sucrose intake by 4.1 and −4.6 g/day, respectively. Furthermore, the IG significantly reduced the consumption of sweets and salty snacks by −0.1 and −0.3 portions/day, respectively. The CG demonstrated a significant increase of body weight and waist circumference by 1.4 kg and 2.1 cm, respectively, while BMI decreased only in the IG by −0.4 kg/m2. These findings indicate that the DST provides significant support to healthcare professionals to favorably modify obesity-related behaviors and BMI in children and adolescents [24].

## 6. Discussion

Overweight and obesity should be perceived as chronic diseases [28,29]. Several scientific and health organizations have suggested the development of appropriate e-Health applications [29,30] to address the obesity epidemic. To date, there has been little evidence on how clinical practice can be enhanced in order to assist children and their parents achieve and maintain appropriate (for their age and gender) body weight and prevent cardiometabolic risk. Only a few studies have investigated the effectiveness of e-Health applications [31] in weight loss beyond one year. Although there are currently several computerized or e-Health tools designed to promote personalized advice on weight management in children and/or adolescents, the majority of those do not involve health professionals in the implementation process [32].

These applications include the Child Health and Obesity Informatics System (CHOIS), which is a secure system that complies with both the Health Insurance Portability and Accountability Act and the Family Educational Rights and Privacy Act, and integrates large databases in a high performance grid computing environment. This application enables nurses to identify school children at risk for obesity and enroll them in prevention and intervention programs [33]. It is very helpful in terms of providing initial guidance to parents and children, however, it does not include any clinical examination findings.

The HopSCOTCH (Shared-Care Obesity Trial in Children) included the development of another web-based shared-care software that reinforced the collaboration between obesity specialists and general practitioners for the best care of their patients, through the provision of a structured yet efficient approach for weight management, and a mechanism to allow both general practitioners and obesity specialists to record and track patient progress simultaneously. This project was difficult to implement and did not perform well [34].

The Child-Teen Obesity Treatment Service Platform included two patient/parent mobile applications, one web-monitoring service for medical staff, one mobile application for food-craving endurance, and one mobile application for medical examinations. The effectiveness of this program is awaiting verification [35].

The smartphone application Pedia BP is another innovative application, which enables observation of the prevalence of pediatric hypertension among children and adolescents aged 3–18 years. The purpose of this study was to identify the prevalence of elevated blood pressure in children and adolescents and to evaluate any association among BMI, age, and blood pressure. The manual systolic and diastolic blood pressure readings were entered into the application, along with the patient’s gender, age, height and weight. Pedia BP instantly calculated the blood pressure classification of the patient based on percentiles. Quantitative data from the Pedia BP data repository were analyzed using descriptive statistics. Pedia BP was shown to be an effective screening tool to easily classify blood pressure readings on an individual basis, however, it is precise only for measuring blood pressure [36].

The “National Registry for the Prevention and Management of Overweight and Obesity in Childhood and Adolescence” and its continuation “Development of Intelligent Multi-level Information Systems and Specialized Artificial Intelligence Algorithms for Personalized Management of Obesity in Childhood and Adolescence” are unique and innovative web-based e-Health professional applications that offer—through predefined therapeutic algorithms—a designed comprehensive personalized multi-disciplinary intervention program, and help to reduce cardiometabolic risk since they combine information on genetic, endocrinologic and environmental risk factors for childhood obesity. Furthermore, they can be translated into other languages and used in different countries [37]. Finally, they can include programs for the prevention and management of obesity in adulthood or other chronic diseases.

The Big Data against Childhood Obesity consortium brings together thirteen European partners and collects large-scale data, which allow us to draw correct conclusions about the behavioral causes of childhood obesity. For example, project-related efforts have already resulted in the quantification of different rates of ultra-processed food marketing in neighborhoods of Stockholm, Sweden, with more such food advertisements being present around subway stations in areas with lower socioeconomic population profiles [38]. Comprehensive models of the obesity prevalence dependence matrix are being created [39], allowing for the first time the data-driven effectiveness predictions about specific policies on a community and the real-time monitoring of the population response, supported by powerful data visualizations.

Finally, the Decision Support Tool provides personalized advice that helps children and adolescents not only achieve better management of their body weight, but also a higher intake of essential micronutrients. The latter is extremely important because insufficiency of micronutrients in children and adolescents with obesity is usually the result of chronic, low-grade inflammation owing to visceral adipocity [40]. This was evidenced by the significant increase in the dietary intakes of iron, magnesium and zinc observed only in the intervention group, which can correct potential obesity-related nutrient insufficiencies [41] and can subsequently support children’s growth, motor and cognitive function [42,43,44]. In addition, the dietary recommendations provided to children and their parents via the Decision Support Tool, were also aiming to reduce the use of table salt as a means to better control their blood pressure levels [45], another common comorbidity of obesity in children.

The above novel e-Health applications are expected to play an important role in the personalized management of overweight and obesity in childhood and adolescence by supporting clinicians to improve on the quality of medical care that they provide. Hence, they will help prevent the development of cardiomatabolic risk factors, thereby reducing morbidity and mortality in late adolescence and/or adulthood. Furthermore, they will allow public health authorities to evaluate their communities based on their obesity prevalence risk and to take local action, based on objective evidence. It is important to note that the long-term effectiveness of all these e-Health applications for the prevention and management of overweight and obesity in childhood and adolescence requires the support of public health authorities, long-term funding by national authorities and the commitment of pediatricians and general practitioners to enter all children and adolescents into the system [30,46].
